# A narrative review of impacts of apolipoproteins on atherosclerotic coronary plaques

**DOI:** 10.1038/s44325-026-00104-x

**Published:** 2026-02-02

**Authors:** Tatsuya Fukase, Tomotaka Dohi

**Affiliations:** 1 Department of Cardiovascular Biology and Medicine, Juntendo University Graduate School of Medicine, Bunkyo-ku, Tokyo Japan; 2 Department of Cardiology, Hakodate Goryokaku Hospital, Hakodate-shi, Hokkaido Japan; 3 Department of Cardiovascular Preventive Medicine, Yumino Medical, Toshima-ku, Tokyo Japan

**Keywords:** Biochemistry, Cardiology, Diseases

## Abstract

Apolipoproteins are structural components of lipoproteins involved in assembly, enzyme regulation, structural integrity, and receptor binding. Apoprotein(a) forms lipoprotein(a), apolipoprotein A-I drives reverse cholesterol transport, apolipoprotein B reflects atherogenic particle number, and apolipoprotein C-III regulates triglycerides. This review highlights the clinical significance of these apolipoproteins in coronary plaque characteristics detected by imaging modality. Additionally, we summarize the current clinical status of therapeutic agents targeting these apolipoproteins and its future potential.

## Introduction

The impacts of apolipoproteins in vivo may seem more esoteric compared to lipoproteins due to these multifaceted functions; however, a basic appreciation of their functions is essential for not only a deeper understanding of lipid and lipoprotein metabolism, but also considering the risk of atherosclerotic cardiovascular disease (ASCVD), a leading cause of morbidity and mortality worldwide^1^. There have been many reports that lipoproteins represented by low-density lipoproteins cholesterol (LDL-C) strongly relate to development of coronary artery plaques that affects ASCVD occurrence^2,3^; however, apolipoproteins also influence atherosclerotic profiles. In this review, we discuss the clinical significance of apolipoprotein(a) [Apo(a)], apolipoprotein A-I (ApoA-I), apolipoprotein B (ApoB), and apolipoprotein C-III (ApoC-III) among serum apolipoproteins that would be particularly relevant to atherosclerotic coronary plaques. Furthermore, we summarize the insights into novel agents that target specific apolipoproteins to reduce the risk of ASCVD and attenuate the atherosclerotic coronary plaques.

## Methods

**Fig. 1:**
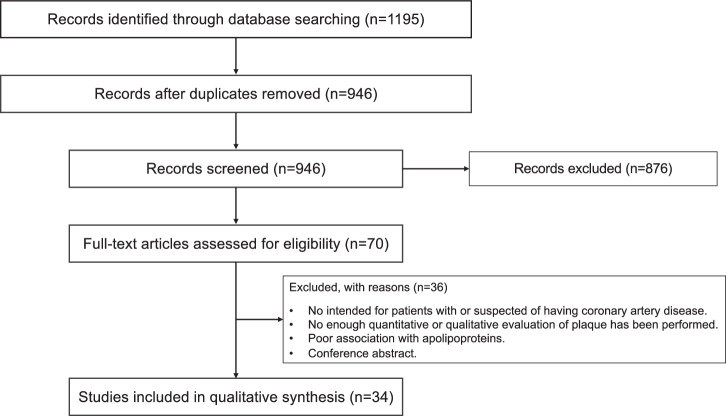
Study selection. PRISMA-style flow diagram illustrating the systematic literature search and selection process. The initial database search yielded 1195 records. After duplicate removal, 946 records were screened at the title and abstract level. Of these, 876 records were excluded. 70 full-text articles were assessed for eligibility, 34 studies finally included in qualitative synthesis.

To be included in this review, studies needed to meet the following criteria; (1) explore apolipoproteins influencing atherosclerotic coronary plaques as a primary aim; (2) qualitative methodology (e.g., data-collection, method of analysis); (3) usage of imaging modality (intravascular ultrasound (IVUS), optical coherence tomography (OCT), and coronary computed tomography angiography (CCTA)) or histopathological examination; (4) focus on adults (i.e., ≥18 years); and (5) published in English. We searched the electronic database of PubMed, SCOPUS, EBSCO, and Cochrane Library from January 2000 until November 2025 for studies that evaluated the association between apolipoproteins and atherosclerotic coronary plaques. The terms used for searching were “*lipoprotein(a)*”, “*apolipoprotein(a)*”, “*apolipoprotein A-I*”, “*apolipoprotein B*”, “*apolipoprotein C-III*”, “*coronary plaques*”, “*intravascular ultrasound*”, “*optical coherence tomography*”, and “*coronary computed tomography angiography*”, and we searched for these combinations. Randomized controlled trials, observational studies, including case control studies, prospective cohort study, retrospective observational study, reviews, and meta-analysis were including if reference was made to apolipoproteins and atherosclerotic coronary plaques. Studies were selected by co-authors by screening the title and abstract. As results, 986 studies were found. Of these, only 34 met the inclusion criteria, and we finally reviewed the full text for the selected studies as shown in Fig. 1. The association of each apolipoprotein with plaques is summarized in Table 1, and section of “Imaging correlates” provided details of major and large clinical studies on the association of each apolipoprotein with atherosclerotic coronary plaques. The unknown confounding factors may have influenced the outcomes despite statical adjustments in each study, and the studies with small sample size may have limited the statistical power.

**Table 1:** Characteristics of the included studies on the association between apolipoproteins and atherosclerotic coronary plaques

Author	Study type	Apolipoproteins	Total subjects	Therapeutic intervention	Results
Huded et al.^42^	Post hoc analysis of 6 randomized controlled trials	Apolipoprotein(a)	3943	PCSK9-mAb Statin ACT Rimonabant CETP inhibitor	PAV measured by IVUS was significantly higher in the high Lp(a) group in unadjusted (38.2% [32.8, 43.6] versus 37.1% [31.4, 43.1], *p* = 0.01) and risk-adjusted analyses (38.7% ± 0.5 versus 37.5% ± 0.5, *p* < 0.001). There was a significant association of increasing risk-adjusted PAV across quintiles of Lp(a) (Lp(a) quintiles 1–5; 37.3 ± 0.5%, 37.2 ± 0.5%, 37.3 ± 0.5%, 38.0 ± 0.5%, 38.5 ± 0.5%, *p* = 0.002).
Koskinas et al.^92^	Randomized controlled trial	Apolipoprotein(a)	265	PCSK9-mAb Statin	In the alirocumab group, the reduction in NIRS-derived maxLCBI_4mm_ was smaller in patients with higher baseline Lp(a), defined by the highest quartile (Q4, ≥98 nmol/L; *n* = 30), than in those with lower baseline Lp(a) (Q1-Q3, <98 nmol/L; *n* = 99; −40.2 [−91.1 to 10.7] versus −91.4 [−113.9 to −68.9], respectively; *p* = 0.01 after adjustment for clinically relevant baseline variables), and was comparable to NIRS-derived maxLBI_4mm_ reduction in the placebo group (−37.60 [−57.40 to −17.80]; *n* = 134).
Puri et al.^55^	Randomized controlled trial	Apolipoprotein(a)	915	Statin	Quartiles of baseline and follow-up Lp(a) did not associate with ΔPAV measured by IVUS. Irrespective of the achieved LDL-C (<vs. ≥70 mg/dL), neither baseline nor on-treatment (<vs. ≥median) Lp(a) levels significantly associated with ΔPAV. No significant differences were observed in ΔPAV in Lp(a) risers versus non-risers, nor in those patients with baseline or on-treatment Lp(a) levels <vs. > 50 mg/dL.
Nozue et al.^52^	Randomized controlled trial	Apolipoprotein(a)	119	Statin	Multivariate logistic regression analysis showed that Lp(a) was a significant independent predictor associated with necrotic core progression detected by VH-IVUS during statin therapy (OR: 3.514; 95% CIs: 1.338–9.228; *p* = 0.01).
Kaiser et al.^43^	Randomized controlled trial	Apolipoprotein(a)	191	DAPT	Patients with high Lp(a) showed accelerated progression of low-attenuation plaque detected by CCTA compared with low Lp(a) patients (26.2 ± 88.4 mm^3^ vs -0.7 ± 50.1 mm^3^; *p* = 0.020). Multivariable linear regression analysis confirmed the relation between Lp(a) and low-attenuation plaque volume progression (*β* = 10.5% increase for each 50 mg/dL Lp(a), 95% CIs: 0.7%–20.3%).
Erlinge et al.^56^	Prospective cohort study	Apolipoprotein(a)	865	N/A	By multivariable analysis, TC, LDL-C, and non-HDL-C (but not Lp[a]) were associated with pancoronary plaque volume and pancoronary LCBI using NIRS-IVUS (*p* < 0.01 for all), but not with the presence of vulnerable plaque. Conversely, Lp(a) (but not TC, LDL-C, or non-HDL-C) was associated with the presence of focal vulnerable plaques (*p* = 0.01).
Matsushita et al.^53^	Prospective cohort study	Apolipoprotein(a)	102	Statin	The low Lp(a) group had significant IVUS-derived plaque regression, whereas the high Lp(a) group showed slight IVUS-derived plaque progression (−6.8% vs. 2.5%, *p* = 0.02).
Lan et al.^44^	Prospective cohort study	Apolipoprotein(a)	246	N/A	The total plaque volume, noncalcified plaque volume, LAP volume, fibro-fatty plaque volume, and fibrotic plaque volume detected by CCTA were higher in the elevated Lp (a) group than in the normal Lp (a) group at baseline (all *p* < 0.001). At follow-up, the elevated Lp (a) group showed a higher mean annual increase in LAP volume than the normal Lp (a) group (3.03 ± 22.26 mm^3^ vs −3.09 ± 12.22 mm^3^; *p* = 0.011).
Nurmohamed et al.^45^	Prospective cohort study	Apolipoprotein(a)	267	N/A	Patients with Lp(a) levels ≥125 nmol/L had twice as high PAV measured by CCTA (6.9% vs 3.0%; *p* = 0.01) compared with patients with Lp(a) levels <125 nmol/L. Adjusted for other risk factors, every doubling of Lp(a) resulted in an additional 0.32% (95% CIs: 0.04–0.60) increment in PAV during the 10 years of follow-up.
Yu et al.^46^	Prospective cohort study	Apolipoprotein(a)	5607	N/A	Elevated Lp(a) levels were associated with myocardial infarction risk (adjusted HR: 1.91; 95% CIs: 1.46–2.49; *p* < 0.001). There was a significant interaction between Lp(a) and LAP detected by CCTA (*P*_interaction_ < 0.001) in relation to myocardial infarction risk. When stratified by the presence or absence of LAP, Lp(a) was associated with myocardial infarction in patients with LAP (adjusted HR: 3.03; 95% CIs: 1.92–4.76; *p* < 0.001). Mediation analysis revealed that LAP mediated 73.3% (Pp < 0.001) for the relationship between Lp(a) and myocardial infarction.
Yu et al.^93^	Prospective cohort study	Apolipoprotein(a)	170	PCSK9-mAb	During follow-up, Lp(a) (12.1 [5.6, 21.8] vs. 18.9 [13.2, 27.2], *p* = 0.002) were reduced after evolocumab treatment. CCTA revealed that the calcified plaque volume were significantly increased (188.3 [115.7, 361.0] vs. 129.3 [59.5, 238.3], *p* = 0.015), while the noncalcified plaque volume and necrotic volume were diminished (107.5 [40.6, 180.6] vs. 125.0 [65.3, 269.7], *p* = 0.038; 0 [0, 4.7] vs. 0 [0, 13.4], *p* < 0.001, respectively). In addition, PCAT density of right coronary artery was significantly attenuated in evolocumab group (−85.0 [−89.0, −82.0] vs. −79.0 [−83.5, −74.0], *p* < 0.001).
Shishikura et al.^54^	Prospective cohort study	Apolipoprotein(a)	439	N/A	Almost one-third of study subjects (33.4%) exhibited both LDL-C < 70 mg/dL and Lp(a) <50 mg/dL. On NIRS imaging analysis, a smaller maxLCBI_4mm_ (*p* < 0.001) and a lower frequency of maxLCBI_4mm_ ≥ 400 (*p* = 0.001) were observed in those with both LDL-C < 70 mg/dL and Lp(a) <50 mg/dL. On multivariable logistic regression analysis, the coexistence of these 2 lipid controls showed an approximately 70% lower risk (adjusted OR: 0.30; 95% CIs: 0.13–0.68) of maxLCBI_4mm_ ≥ 400 compared with the reference group (LDL-C ≥ 70 mg/dL and Lp(a) ≥50 mg/dL).
Wang et al.^49^	Retrospective observational study	Apolipoprotein(a)	177	N/A	Multivariable linear regression model revealed an association between Lp(a) and OCT-derived plaque progression in non-culprit atherosclerosis (7.22 for each 1 SD increase, 95% CIs: 0.96–13.48; *p* = 0.025) and increased lipid component (3.60 for each 1 SD increase, 95% CIs: 0.63–6.56; *p* = 0.019).
Yang et al.^47^	Retrospective observational study	Apolipoprotein(a)	1052	N/A	Patients experiencing major adverse cardiovascular events demonstrated elevated Lp(a) levels and PACT attenuation values measured by CCTA (26.20 (17.63–45.08) vs. 12.00 (4.58–26.00), *p* < 0.001; −72.6 ± 8.4 HU vs. −79.7 ± 9.5 HU, *p* < 0.001). Lp(a) was associated with PCAT attenuation (*p* < 0.001), and elevated PCAT attenuation was associated with quantitatively greater atherosclerotic burden (*p* < 0.001).
Di Muro et al.^50^	Cross-sectional study	Apolipoprotein(a)	202	N/A	OCT findings revealed that patients with elevated Lp(a) had a higher prevalence of lipid-rich plaques, a significantly greater mean lipid arc, along with increased macrophage infiltration and TCFA. Multivariable regression analysis identified LDL-C ≥ 70 mg/dL, and elevated Lp(a) as strong predictors of lipid-rich plaques.
Niccoli et al.^51^	Cross-sectional study	Apolipoprotein(a)	51	N/A	In the OCT cohort, patients with higher Lp(a) levels (≥30 md/dl) compared to patients with lower Lp(a) levels (<30 md/dl) exhibited a higher prevalence of lipidic plaque at the site of the culprit stenosis (67% vs. 27%; *p* = 0.02), a wider lipid arc (135 ± 114 vs 59 ± 111; *p* = 0.03) and a higher prevalence of TCFA (38% vs. 10%; *p* = 0.04).
Hikita et al.^48^	Cross-sectional study	Apolipoprotein(a)	68	N/A	CCTA indicated that the group with an Lp(a) level of 25 mg/dL or more had a greater number of total plaques, noncalcified plaques, and low-attenuation plaques in whole coronary arteries than did the group with an Lp(a) level of less than 25 mg/dl (5.3 ± 1.8 vs. 3.7 ± 2.2, *p* = 0.0061; 4.0 ± 2.0 vs. 1.2 ± 1.3, *p* = 0.0001; 2.2 ± 2.1 vs. 0.5 ± 0.7, *p* = 0.0001, respectively).
Solem et al.^57^	Randomized controlled trial	Apolipoprotein A-I	22	Statin	The histopathological examination revealed that plasma levels of ApoA-I correlated positively with tissue collagen and inversely with metalloproteinase-9 and macrophage content in patients with stable angina and coronary plaques suitable for directional coronary atherectomy.
Mani et al.^59^	Prospective cohort study	Apolipoprotein A-I	2566	Statin	Increasing levels of achieved HDL-C/ApoA-I ratio (*p* = 0.04), but not HDL-C (*p* = 0.18) or ApoA-I (*p* = 0.67), were associated with less progression of PAV measured by IVUS. Similar results were seen for change in total atheroma volume, with less progression seen with increased HDL-C/ApoA-I ratio (*p* = 0.002) but not with increases in HDL-C (*p* = 0.09) or ApoA-I (*p* = 0.19).
Suruga et al.^61^	Cross-sectional study	Apolipoprotein A-I	297	N/A	The multivariate logistic analysis demonstrated that oxHDL/ApoA-I ratio significantly associated with the presence of high-risk plaques and significant coronary stenosis detected by CCTA (*p* = 0.01 and < 0.01).
Bamberg et al.^58^	Cross-sectional study	Apolipoprotein A-I	313	N/A	High-sensitivity C-reactive protein and ApoA-I were associated with the extent of non-calcified plaque detected by CCTA (*p* = 0.03 and *p* = 0.004, respectively).
Funabashi et al.^109^	Randomized controlled trial	Apolipoprotein B	78	Statin	Both ApoB levels and NIRS-IVUS images at baseline and week 48 were analyzed in type 2 diabetic patients. There was no significant difference in the atheroma progression rate between both groups (−0.27 ± 0.15% vs −0.33 ± 0.51%, *p* = 0.44); however, patients with any reduction of ApoB levels exhibited a greater frequency of change in maxLCBI_4mm_ (−13.4 ± 22.2% vs 70.3 ± 28.7%, *p* = 0.03) and maxLCBI_4mm_ regression (61.1 ± 0.08% vs 31.0 ± 0.09%, *p* = 0.02).
Fujino et al.^111^	Randomized controlled trial	Apolipoprotein B	112	PCSK9-mAb Statin	Coronary plaque phenotypes in patients with non-ST elevation myocardial infarction treated with evolocumab plus statin or placebo plus statin for 52 weeks using OCT. Patients achieving the ApoB goal (<65 mg/dL) demonstrated a greater increase in minimum fibrous cap thickness (+44.6 ± 36.0 vs +24.9 ± 38.1 μm, *p* = 0.007) and a more pronounced decrease in lipid arc (−57.8 ± 52.8 vs −27.0 ± 59.2°, *p* = 0.005) at follow-up, compared with those who did not achieve the ApoB goal.
Arai et al.^110^	Prospective cohort study	Apolipoprotein B	251	Statin	Decreasing ApoB quartiles were associated with a progressively smaller plaque burden measured by IVUS in total and type 2 diabetic patients (*p* = 0.006 and *p* = 0.049, respectively). In type 2 diabetic patients, further reduction of these parameters was associated with a significantly greater reduction in plaque volume.
Bayturan et al.^65^	Prospective cohort study	Apolipoprotein B	3437	Statin	Multivariable analysis revealed that independently associated risk factors of progression in patients with LDL-C ≤ 70 mg/dL included baseline IVUS-derived PAV (*p* = 0.001), presence of diabetes mellitus (*p* = 0.02), increase in systolic blood pressure (*p* = 0.001), less increase in HDL-C (*p* = 0.01), and a smaller decrease in ApoB levels (*p* = 0.001).
Voros et al.^66^	Prospective cohort study	Apolipoprotein B	60	N/A	ApoB particles were associated with a higher proportion of noncalcified plaque and a lower proportion of calcified plaque (small dense LDL-C and high-density noncalcified plaque: *r* = 0.3, *p* = 0.03; triglycerides and low-density noncalcified plaque: *r* = 0.34, *p* = 0.01) using VH-IVUS.
Ohwada et al.^67^	Cross-sectional study	Apolipoprotein B	115	N/A	The %necrotic core volume evaluated by VH-IVUS correlated with LDL-C (*r* = 0.2353, *p* = 0.0114) and ApoB (*r* = 0.2487, *p* = 0.0074) but not with HDL-C and ApoA-I.
Tani et al.^68^	Prospective cohort study	Apolipoprotein B/A-I ratio	64	Statin	The plaque volume, assessed by IVUS, had decreased significantly by 12.6% (*p* < 0.0001, vs baseline), and a significant decrease of 6.4% and 14.6% was found in the serum level of ApoB and the ApoB/A-I ratio (*p* = 0.0001 and *p* < 0.0001, respectively, vs baseline) A stepwise regression analysis revealed that the change in the ApoB/A-I ratio was an independent predictor of the change in coronary plaque volume (β coefficient 0.386; *p* = 0.0023).
Deng et al.^69^	Cross-sectional study	Apolipoprotein B/A-I ratio	320	N/A	The high ApoB/A-I ratio was associated with high percent of vulnerable plaques detected by OCT compared with low ratio group. The ApoB/A-I ratio was negatively related to fibrous cap thickness in lipid-rich plaque (*r* = −0.228, *p* = 0.043).
Du et al.^70^	Cross-sectional study	Apolipoprotein B/A-I ratio	199	N/A	Multivariate logistic regression analysis revealed that both ApoB/A-I ratio (OR: 1.041, 95% CIs: 1.007–1.076; *p* = 0.019) and TCFA (OR: 3.199, 95% CIs: 1.133–9.031; *p* = 0.028) were significantly related to high tissue prolapse volume detected by OCT.
Jung et al.^71^	Cross-sectional study	Apolipoprotein B/A-I ratio	1401	N/A	After adjustment for confounding variables, each 0.1 increase in serum ApoB/A-I was significantly associated with increased odds ratios for coronary stenosis and non-calcified plaques detected by multidetector computed tomography of 1.23 and 1.18, respectively.
Kitahara, et al.^80^	Randomized controlled trial	Apolipoprotein C-III	78	Statin	Both ApoC-III levels and NIRS-IVUS images at baseline and week 48 were analyzed in type 2 diabetic patients. Serial changes in IVUS-derived atheroma volume were similar between two groups (−0.7 ± 2.2 vs. −2.4 ± 1.6 mm^3^, *p* = 0.51); however, greater progression in NIRS-derived maxLCBI_4mm_ was observed in those with any increase in ApoC-III levels (91.2 ± 24.8 vs. −44.2 ± 23.5, *p* < 0.001).
Qamar et al.^81^	Cross-sectional study	Apolipoprotein C-III	1422	N/A	In type 2 diabetic patients, increased plasma ApoC-III is associated with higher coronary artery calcification detected by CCTA (tobit regression ratio, 1.78; 95% confidence interval, 1.27–2.50 per SD increase in ApoC-III; *p* < 0.001).
Fukase et al.^82^	Cross-sectional study	Apolipoprotein C-III	202	N/A	Multivariable logistic regression analysis showed that the highest ApoC-III quartile was significantly associated with severe calcification and calcified nodules detected by IVUS, with the lowest ApoC-III quartile as the reference (OR: 2.70, 95% CIs: 1.04–7.00, *p* = 0.042; and OR: 3.72, 95% CIs: 1.26–11.0, *p* = 0.017, respectively). ApoC-III level (1-mg/dl increase) was a strong significant predictor of severe calcification (OR: 1.07, 95% CIs: 1.00–1.15, *p* = 0.040) and calcified nodules (OR: 1.09, 95% CIs: 1.01–1.19, *p* = 0.034) according to the multivariable logistic regression analysis.

*ACT* adoptive cell therapy, *ApoA-I* apolipoprotein A-I, *ApoB* apolipoprotein B, *ApoC-III* apolipoprotein C-III, *ApoE* apolipoprotein E, *CCTA* coronary computed tomography angiography, *CETP* cholesteryl ester transfer protein, *CIs* confidence intervals, *DAPT* dual anti-platelet therapy, *HDL-C* high-density lipoprotein cholesterol, *HR* hazard ratio, *IVUS* intravascular ultrasound, *LAP* low attenuation plaque, *LCBI* lipid core burden index, *LDL-C* low-density lipoprotein cholesterol, *Lp(a)* lipoprotein (a), *MaxLCBI*_4mm_ maximum lipid core burden index calculated for every 4-mm segment, *NIRS* near-infrared spectroscopy, *OCT* optical coherence tomography, *OR* odds ratio, *oxHDL* oxidized high-density lipoprotein, *PAV* percentage atheroma volume, *PCAT* pericoronary adipose tissue, *PCSK9-mAb* proprotein convertase subtilisin/kexin 9 monoclonal antibody, *SD* standard deviation, *TC* total cholesterol, *TCFA* thin-cap fibroatheroma, *VH-IVUS* virtual histology intravascular ultrasound.

### Pathophysiological roles and genetic insights

**Fig. 2:**
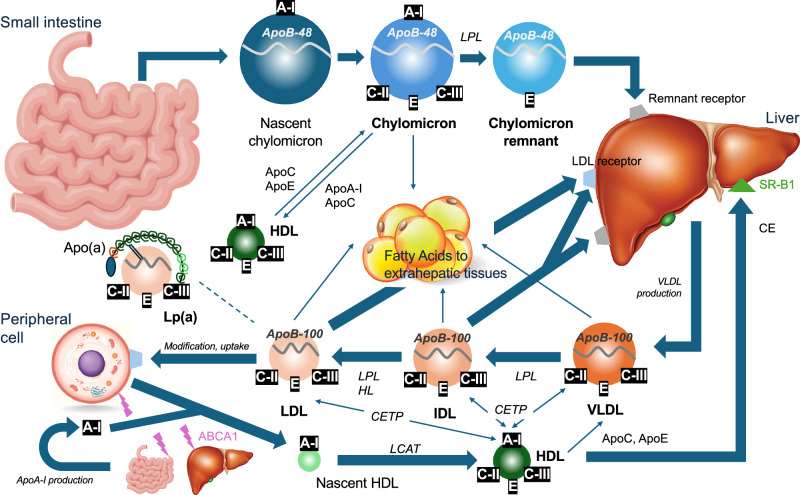
Regulation of lipoproteins and apolipoproteins metabolism. In the small intestine, chylomicrons are assembled to transport dietary fats from the enterocytes into the lymphatic system, which subsequently then delivers them to the bloodstream. Dietary lipids are digested, re-esterified, and packaged together with apolipoproteins such as ApoB-48, ApoC-II, ApoC-III, and ApoE into chylomicrons. These particles distribute fatty acids and fat-soluble vitamins to peripheral tissues before their remnants are cleared by the liver. The endogenous lipoprotein pathway begins in the liver with the secretion of VLDL. TG carried in VLDL are hydrolyzed in muscle and adipose tissue by LPL, releasing free fatty acids and generating IDL. IDL particles are further metabolized to by LPL and HL to form LDL, which delivers cholesterol to peripheral tissues and is cleared primarily by LDL receptors in the liver. Lp(a) is structurally similar to LDL, consisting of ApoB-100 covalently bound to Apo(a), but functions independently and is recognized as a distinct atherogenic lipoprotein. Reverse cholesterol transport is the pathway by which excess peripheral cholesterol is returned to the liver. ApoA-I, secreted by the liver and intestine, acquires free cholesterol from peripheral cells, including macrophages and endothelial cells, via ABCA1, forming the nascent HDL. This nascent HDL is converted into mature HDL by LCAT, which esterifies free cholesterol into CE that are sequestered into the particle core. HDL delivers cholesterol to the liver either directly, through interaction with hepatic SR-B1, or indirectly, via CETP-mediated exchange of TG with other atherogenic lipoproteins (chylomicron, VLDL, IDL, and LDL). ABCA1 ATP-binding cassette transporter A1, ApoA-I apolipoprotein A-I, Apo(a) apolipoprotein (a), ApoB-48 apolipoprotein B-48, ApoB-100 apolipoprotein B-100, ApoC-II apolipoprotein C-II, ApoC-III apolipoprotein C-III, ApoE apolipoprotein E, CE cholesterol ester, CETP cholesteryl ester transfer protein, HDL high-density lipoprotein, HL hepatic lipase, IDL intermediate-density lipoprotein, LCAT lecithin: cholesterol acyltransferase, LDL low-density cholesterol, Lp(a) lipoprotein (a), LPL lipoprotein lipase, SR-B1 scavenger receptor B1, TG triglyceride, VLDL very low-density lipoprotein.

Apolipoproteins are essential components of plasma lipoproteins, which have complex macromolecular structures composed of an envelope of phospholipids and free cholesterol, and a core of cholesteryl ester and triglyceride (TG). The term “apolipoprotein” is made up of two words: “apo,” a Greek word that means “away from,” and “lipoprotein,” which refers to the lipid-protein complex^4^. Clinically important apolipoproteins such as apolipoprotein ApoA-I, apolipoprotein A-II, apolipoprotein B-100 (ApoB-100), apolipoprotein B-48 (ApoB-48), apolipoprotein C-II (ApoC-II), ApoC-III, apolipoprotein E (ApoE), and Apo(a) are associated with several disease conditions, including dyslipidemia, cardiovascular disease and neurodegenerative disorders. Figure 2 shows the illustrative diagram of lipid transport and metabolic pathway. Among them, ApoB is the only non-exchangeable apolipoprotein; it’s β-sheet secondary structure enables stable binding with lipoprotein particles^5^. The other apolipoproteins are exchangeable, and they can dissociate from one lipoprotein and reassociate with another lipoprotein^6^. Apolipoproteins have four major roles: (1) assembly and secretion of the lipoprotein (ApoA-I, ApoB-100, and ApoB-48)^7,8^; (2) coactivators or inhibitors of enzymes (ApoA-I, ApoC-II, and ApoC-III)^9,10^; (3) structural integrity of lipoprotein (ApoB, ApoE, and ApoA-I)^11^; and (4) binding or docking to specific receptors and proteins for cellular uptake of the entire particle or selective uptake of the lipid component (ApoA-I, ApoB-100, and ApoE)^11^. Among these, we will highlight and review apolipoproteins, which are thought to be closely related to atherosclerotic coronary plaques. The structure, function, and pathophysiological role in ASCVD of each apolipoprotein are summarized in Table 2.

**Table 2:** Characteristics of the included studies on the association between apolipoproteins and atherosclerotic coronary plaques

Apolipoprotein	Genes	Isoforms	Synthesis	Structure	Function	Lipoproteins	Pathophysiological role in ASCVD
Apolipoprotein(a)	4529	Over 20 isoforms, dependent on number of kringle 4 repeats	Liver	Variable molecular mass: 187,000–800,000 DaPre-beta mobility High sialic acid content	Binds to immobilized fibronectin and endows Lp(a) with the serine-proteinase-type proteolytic activity	LDL, HDL_2_	High Lp(a) levels are associated with accelerated progression of low-attenuation atherosclerotic plaques in patients with coronary artery disease, contributing to residual myocardial infarction risk despite treatment with lipid-lowering therapy. Additionally, it has reported that Lp(a) activates monocytes, promoting inflammation and thrombosis, suggesting a direct role in increasing immuno-thrombotic risk in patients with ASCVD^125^. Furthermore, ApoB-based genetic analyses indicate that, on a per particle basis, Lp(a) is approximately six times more atherogenic than LDL^126^. Numerous studies report that elevated Lp(a) contribute to residual cardiovascular risk in patients who underwent percutaneous coronary intervention^127,128^.
Apolipoprotein A-I	Chromosome 11, A1/C3/A4/A5 gene cluster	Six polymorphic isoforms. Mutations: ApoA-I Tangier, ApoA-I Milano, ApoA-I Marburg	Liver, intestine	243 AA 28,000 Da	Activates LCAT Interacts with ABCA1 for initial lipidation of HDL during HDL biogenesis Interacts with SR-B1 foe selective lipid uptake and cholesterol efflux Pro-inflammatory in the absence of ApoE	70% of HDL protein. Most abundant protein in HDL. Chylomicrons, VLDL	HDL particles can be divided into subclasses based on physicochemical properties: HDL2, which is larger and more buoyant, and HDL3, which is smaller and denser. HDL3 typically contains both ApoA-I and ApoA-II, whereas HDL2 contains only ApoA-I^129^. It has been reported that worse cardiovascular outcomes were more strongly associated with HDL3 rather than HDL2 in patients with ASCVD; thus, the role of ApoA-I as a biomarker of cardiovascular risk could be evaluated^130^. Our facility registry has demonstrated that the low ApoA-I group had a significantly higher cumulative incidence rate of cardiovascular events than the high ApoA-I group^131^. It might be related to the fact that high CEC has been inversely related to the incidence of ASCVD events^132^.
Apolipoprotein B	Chromosome 2	ApoB-100: Over 100 polymorphisms	ApoB-100: Liver ApoB-48: Intestine	ApoB-100: 4536 AA, 550,000 Da ApoB-48: 2152 N-terminal AA of B100, 264,000 Da. 8–10% carbohydrates	ApoB-100: Acts as a ligand for the LDL receptor In the liver, it promotes the formation of nascent VLDL ApoB-48: In the intestine, it promotes the formation of chylomicron	ApoB-100: VLDL, IDL, LDL ApoB-48: Chylomicron, chylomicron remnant	These analyses have resulted in discordant subgroups, representing approximately 20% to 60% of participants, in whom LDL-C and ApoB levels are not aligned, and ApoB or LDL particle number was a stronger predictor of cardiovascular risk compared to LDL-C. Furthermore, ApoB was shown to be a more accurate marker of cardiovascular risk compared to non-HDL-C^133–137^. A prospective study reported that ApoB was strongly associated with fatal myocardial infarction in both men and women, and was a stronger risk predictor than LDL-C^72^. Thus, ApoB could a more accurate index of the adequacy of LDL lowering therapy than LDL-C.
Apolipoprotein C-III	Chromosome 11, apolipoprotein A1/C3 A4/A5 gene cluster	Variants with differing sialic acid content: CIII-0, CIII-1, CIII-2	Liver, intestine	79AA, 8800 Da	Inhibits LPL activity Promotes the de novo biogenesis of HDL independently of ApoA-I	Surface of TG-rich particles: chylomicrons, VLDL, remnants HDL	The epidemiological studies indicate that elevated ApoC-III levels are associated with increased ASCVD risk and cardiovascular mortality^138–140^. Additionally, the ARIC study demonstrated that elevated LDL-TG, ApoC-III, and ANGPTL3 levels were associated with coronary heart disease and ASCVD events, and concluded that evaluating the potential benefit of lowering hepatic ApoC-III or ANGPTL3 expression in patients with elevated TRL may be clinically important^141^.

*ABCA1* ATP-binding cassette transporter A1, *ApoA-I* apolipoprotein A-I, *ApoA-II* apolipoprotein A-II, *ApoB* apolipoprotein B, *ApoB-48* apolipoprotein B-48, *ApoB-100* apolipoprotein B-100, *ApoC-III* apolipoprotein C-III, *ApoE* apolipoprotein E, *ASCVD* atherosclerotic cardiovascular disease, *CEC* cholesterol efflux capacity, *HDL* high-density lipoprotein, *IDL* intermediate-density lipoprotein, *LCAT* lecithin: cholesterol acyltransferase, *LDL* low-density lipoprotein, *LDL-C* low-density lipoprotein cholesterol, *Lp(a)* lipoprotein (a), *LPL* lipoprotein lipase, *SR-B1* scavenger receptor B1, *TG* triglyceride, *VLDL* very low-density lipoprotein.

**Fig. 3:**
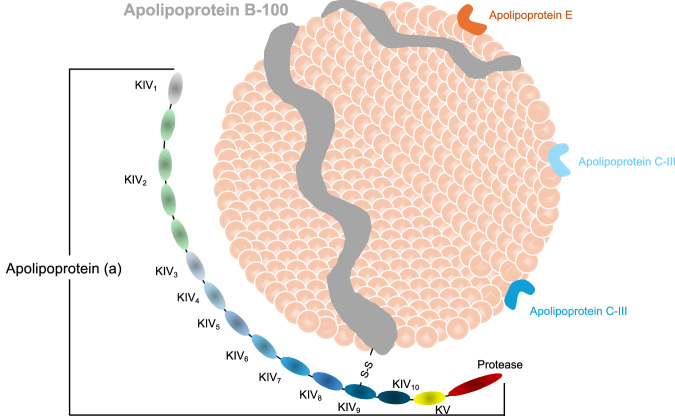
Lipoprotein (a) structure. Lp(a) is composed of two parts; LDL-like particles with ApoB-100 and Apo(a) covalently bound by disulfide bounds. Apo(a) contains 10 types of KIV subtypes: one copy of KIV1 and KIV3-10, and variable KIV2 repetition. Apo(a) apolipoprotein (a), ApoB-100 apolipoprotein B-100, KIV kringle IV, LDL low-density cholesterol, Lp(a) lipoprotein (a).

Circulating lipoprotein (a) [Lp(a)] levels are primarily determined by the *LPA* gene locus^12^. Apo(a) is synthesized almost exclusively in the liver, but the site of assembly of Lp(a) has not been confirmed, and may be within the hepatocyte, the space of Disse, or the plasma compartment^13^. Lp(a) assembly begins with Apo(a) docking to low-density lipoprotein (LDL), followed by formation of a covalent disulfide bond between Kringle IV-type 9 of Apo(a) and ApoB of LDL, as shown in Figs. 2 and 3. The LDL component is thought to be derived from a newly synthesized ApoB-100^14^. *LPA* contains multiple Kringle domains, including a hypervariable Kringle IV-type 2 region of ~5500 bp, which occurs in tandem from fewer than six copies to at least 40 times, and higher KIV-2 copy number generally associates with decreased Lp(a) protein abundance^15,16^. Lp(a) levels are determined almost exclusively by variants in the *LPA* gene, without significant dietary or environmental influences; thus, Lp(a) measurement is recommended at least once in a lifetime in several guidelines^17,18^. Lp(a) levels in humans are primarily measured by immunoassays using polyclonal antibodies against Apo(a). There are two common approaches for reporting results. The first is based on the assignment of target values to the assay calibrators in terms of total Lp(a) mass (Apo(a), ApoB and the lipid components). The values are expressed in mg/dL and there is no traceability of the various calibrators to any established reference material. The second approach, used in several commercial methods, is to assign the target values to assay calibrators traceable to the World Health Organization and the International Federation of Clinical Chemistry and Laboratory Medicine secondary reference materials. The values are expressed in nmol/L of Apo(a), reflecting the number of circulating particles rather than the variable mass of Apo(a) or lipid component^19^. The assay calibration in mg/dL of total Lp(a) mass should be discontinued considering that only Apo(a) is measured by the antibodies and that the mass of Apo(a) is highly variable; thus, it is recommended that standardized Lp(a) assays report values in Apo(a) particle number, as nmol/L.

**Fig. 4:**
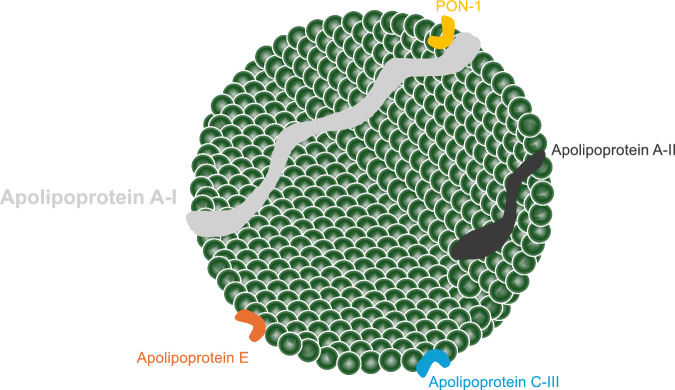
High-density lipoprotein structure. ApoA-I is the major protein constituent of HDL, playing a crucial structural and functional role. PON1 is present in a small fraction of HDL, and its antioxidant activity strictly depends on its binding to ApoA-I. ApoA-I apolipoprotein A-I, HDL high-density lipoprotein, PON1 paraoxonase 1.

ApoA-I encoded by *APOA1* is a protein composed of 243 amino acids with a molecular weight of approximately 28,400 Da, and is synthesized and secreted by the small intestine and liver^20^. ApoA-I is a major structural and functional protein component of high-density lipoprotein (HDL) as shown in Fig. 4, constituting approximately 70% of its composition^21^. Through this role, ApoA-I is central to lipid metabolism, mediating cholesterol transport, and inflammatory, immunological, and vasodilatory pathways^22^. In particular, ApoA-I is crucial for HDL maturation, and two sequential lipidation steps are required, shown in Fig. 2^20^. ATP-binding cassette transporter A1 (ABCA1) loads ApoA-I with phospholipids and cholesterol to form nascent HDL. Nascent HDL incorporating ApoA-I is secreted into the blood, and then matured by lecithin: cholesterol acyltransferase (LCAT). The most important function of ApoA-I is the activation of LCAT, an enzyme that converts free cholesterol on lipoproteins to cholesterol ester (CE). This reaction is essential for the maturation of nascent HDL and for the esterification of cholesterol derived from peripheral tissues. The rapid CE accumulation and phospholipid transfer convert the lipid-poor discoidal HDL into CE-rich spherical HDL particles; however, defects in either ABCA1-mediated lipidation or LCAT activity result in reduced HDL ApoA-I levels^23,24^. Mature HDL particles mediate cholesterol efflux from foam cells after interacting with ATP-binding cassette transporter G1/4 and scavenger receptor B1 (SR-B1). Subsequently, cholesterol-laden mature HDL delivers CE to the liver via hepatic SR-B1^25,26^. Therefore, ApoA-I, the main carrier protein of HDL, is critical to reverse the cholesterol transport process which is the pathway by which excess peripheral cholesterol is returned to the liver, and plays a crucial role in the cellular cholesterol efflux capacity (CEC) mechanism.

**Fig. 5:**
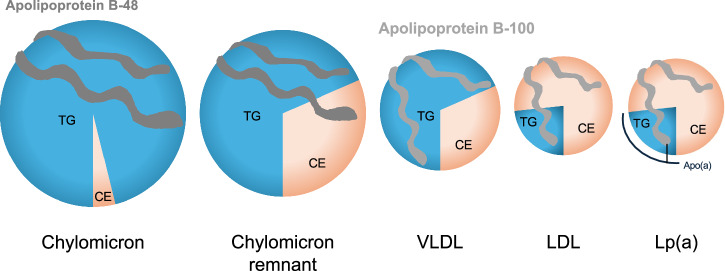
Schematic diagram of apolipoprotein B-48 containing and apolipoprotein B-100 containing lipoproteins. One molecule of ApoB-100 encircles VLDL, LDL, and Lp(a) particles; whereas, one molecule of ApoB-48 encircles a chylomicron or chylomicron remnant particles. Apo(a) apolipoprotein (a), ApoB-48 apolipoprotein B-48, ApoB-100 apolipoprotein B-100, CE cholesterol ester, LDL low-density cholesterol, Lp(a) lipoprotein (a), TG triglyceride, VLDL very low-density lipoprotein.

ApoB is an essential component of very low-density lipoprotein (VLDL), and its metabolites, IDL and LDL as shown in Fig. 5, as well as chylomicron and their remnants^27^. The ApoB particle serves as a structural scaffold, crucial for lipoprotein stability^28^. There are two circulating forms of ApoB. ApoB-100 is mainly synthesized and expressed in the liver and is an integral component of VLDL, IDL and LDL^29^, whereas ApoB-48 together with ApoC-II, ApoC-III, and ApoE is primarily synthesized and expressed within the small intestine and is present in chylomicron and its remnant, shown in Fig. 2^30^. Both ApoB-100 and ApoB-48 are encoded by *APOB*. ApoB-100 consists of 4536 amino acids (molecular weight ~540 kDa), while ApoB-48 consists of 2152 amino acids (molecular weight ~264 kDa)^31^. ApoB-48, found on chylomicron and their remnants, is cleared primarily by the heparin sulfate proteoglycan pathway because they lack an LDL-receptor (LDLR) biding domain^32^. Elevated ApoB-48 levels may result from obesity, diabetes mellitus, hypertriglyceridemia, genetic disorders, or high-fat diets. ApoB-48 is unique to intestinal chylomicron-derived remnant cholesterol particles and contribute to non-fasting remnant cholesterol concentration^33^. While chylomicron itself is too large to penetrate the arterial wall, its remnant may do so and is thought to contribute to lipid accumulation in atherosclerotic plaque^34^. In addition, fasting ApoB-48 is associated with the incidence of coronary artery disease, and elevated ApoB-48 levels, together with risk factors for metabolic syndrome, exacerbate the risk of coronary artery disease^35^; however, of the two forms, ApoB-100 is more clinically relevant in determining the level of circulating atherogenic lipoproteins^36^. ApoB measurement can be considered a powerful tool for assessment of atherogenic lipid status, such as VLDL cholesterol, IDL cholesterol, LDL-C, and Lp(a) particle because each particle contains exactly one molecule of ApoB-100. Nascent ApoB-100 particles are lipidated in the endoplasmic reticulum by microsomal TG transfer protein to form TG-rich VLDL particles. After they are secreted into the circulation, lipoprotein lipase (LPL) metabolizes VLDL to produce IDL, which is further metabolized by LPL and hepatic lipase to form cholesteryl ester-rich LDL. LDL carries the majority of the circulating cholesterol, and can be oxidatively modified and taken up by macrophages which leads to excess accumulation and the formation of foam cells^37^. As such, more than 90% of circulating ApoB resides in LDL particles^38^.

LPL, synthesized in adipocytes, skeletal myocytes, and cardiomyocytes, is an essential enzyme for the lipolytic processing of TG in chylomicron and VLDL. ApoC-II is a critical LPL cofactor that promotes TG hydrolysis by facilitating substrate entry into the enzyme’s active site, whereas ApoC-III and angioproietin-related protein 3 (ANGPTL3) function as an inhibitor^39^. ApoC-III is primarily synthesized by the liver and to a lesser extent by enterocytes, where the human *APOC3* gene is expressed. Mature ApoC-III protein consists of 79 amino acids, with a molecular mass of 8.8 kDa^40^. Recent research has demonstrated that ApoC-III impacts TG-rich lipoproteins (TRL) metabolism, inflammation, atherosclerosis progression, glucose metabolism, and cardiovascular diseases^41^. ApoC-III inhibits hepatic uptake of TRL remnants uptake by displacing ApoE from the lipoprotein surface. This prevents ApoE from interacting with the LDLR and LDL-related protein 1 on hepatocytes. Consequently, ApoC-III influences atherosclerosis by promoting LDL retention and aggregation in the subendothelial space, as well as triggering inflammatory cascades and smooth muscle cell proliferation in the arterial wall^39^.

### Imaging correlates

Our search yielded multiple studies with divergent conclusions as shown in Table 1. We finally selected 17 clinical studies on the association between Lp(a) levels and atherosclerotic coronary plaques. LDL and remnant enter the intima and become trapped partly due to pressure gradient and their binding to glycosaminoglycans. Both lipoproteins can be internalized by macrophages to produce foam cells, which are a principal component of early atherosclerotic lesions. Lp(a) similarly enters to the intima; however, it remains unclear whether macrophages internalized Lp(a) to form foam cells. High plasma levels of LDL, remnants and possibly Lp(a) promote accumulation of inflammatory cells, including foam cells, enhancing atherosclerotic plaque formation. As a major study on the Lp(a) and coronary plaques detected by IVUS, post hoc analysis of 6 randomized controlled trials was divided into the two groups; 683 subjects (17.3%) had Lp(a) ≥ 60 mg/dL and 3260 subjects (82.7%) had Lp(a) < 60 mg/dL, and showed that percentage atheroma volume (PAV) measured by IVUS was significantly higher in the high Lp(a) group in unadjusted (38.2% [32.8, 43.6] vs. 37.1% [31.4, 43.1], *p* = 0.01) and risk-adjusted analyses (38.7% ± 0.5 vs. 37.5% ± 0.5, *p* < 0.001). There was a significant association of increasing risk-adjusted PAV across quintiles of Lp(a) (Lp(a) quintiles 1–5; 37.3 ± 0.5%, 37.2 ± 0.5%, 37.3 ± 0.5%, 38.0 ± 0.5%, 38.5 ± 0.5%, *p* = 0.002)^42^. In addition, some clinical studies have reported that elevated baseline Lp(a) levels in patients with ASCVD are associated with accelerated plaque progression assessed by CCTA^43–48^, high-risk plaque features detected by OCT^49–51^, and plaque burden and necrotic core progression on IVUS^42,52,53^. Additionally, Shishikura, et al. has reported that LDL-C levels and Lp(a) levels are independent strong risk factors for large lipid-rich plaques detected by near-infrared spectroscopy (NIRS)-IVUS. Patients with both LDL-C < 70 mg/dL and Lp(a) < 50 mg/dL have an approximately 70% lower risk of large lipid-rich plaques compared with patients with both LDL-C ≥ 70 mg/dL and Lp(a) ≥50 mg/dL, at the de-novo target lesion level^54^. However, the SATURN has shown that Lp(a) levels (predominantly below the 50 mg/dL threshold) do not associate with coronary atheroma progression^55^. It may occur that the 60% of patients in SATURN were already taking maximally intensive statin therapy, so there is a possibility that regression of focal large vulnerable plaque may have been potentially achieved. In addition, this may be due to the fact that the plaque burden reported in SATURN was tended to be smaller compared with other studies^42,52,53^. The PROSPECT II substudy has recently revealed that LDL-C were associated with pancoronary plaque volume and lipid core; conversely, Lp(a) was associated with the presence of focal large plaque burden and vulnerable plaque^56^. In summary, the difference in their effects on plaque localization demonstrates that Lp(a) may pose a residual risk not only at the patient level but also at the vessel level in an era when LDL-C lowering therapy is the mainstay of ASCVD treatment. Thus, therapeutic intervention targeting Lp(a) would be valid at strict LDL-C lowering therapy-treated patients with low LDL-C and high Lp(a).

As for the anti-atherosclerotic properties of apolipoproteins, some studies have reported on the relationship between ApoA-I and coronary plaques. A histopathological study revealed that ApoA-I levels correlated positively with tissue collagen and inversely with metalloproteinase-9 and macrophage contents in patients stable angina who underwent directional coronary atherectomy^57^. Also, ApoA-I levels were associated with less progression of atheroma volume^58^. As a prospective study on the ApoA-I and coronary plaques detected by IVUS showed that increasing levels of achieved HDL-C/ApoA-I ratio (*p* = 0.04), but not HDL-C (*p* = 0.18) or ApoA-I (*p* = 0.67), were associated with less progression of PAV measured by IVUS. Similar results were seen for change in total atheroma volume, with less progression seen with increased HDL-C/ApoA-I ratio (*p* = 0.002) but not with increases in HDL-C (*p* = 0.09) or ApoA-I (*p* = 0.19)^59^. In a basic research, it has been reported that poor regression of atherosclerotic plaques after LDL-C lowering in mice with diabetes mellitus can be overcome by raising levels of HDL particles by increasing expression of ApoA-I^60^. Whereas, oxidized HDL, which exhibited reduced CEC and anti-inflammatory properties, has been linked to high-risk plaques and significant stenosis detected by CCTA, as a marker of oxidized HDL/ApoA-I ratio^61^. HDL and ApoA-I recovered from human atheroma are dysfunctional and are extensively oxidized by myeloperoxidase. Myeloperoxidase accumulates within the subendothelial compartment, and mechanistically links to the formation of vulnerable plaques by mechanisms including catalytic consumption of nitric oxide, activation of matrix metalloproteinase pathways, and promotion of endothelial cell apoptosis and superficial erosions within culprit lesions^62^.

All ApoB-containing lipoproteins <70 nm in diameter, including Lp(a), LDL, smaller TRL, and their remnant particles, can cross the endothelial barrier, especially in the presence of endothelial dysfunction, where they can become trapped by interactions with extracellular structures such as proteoglycans^63^. Atherosclerotic plaques grow over time as ApoB-containing lipoprotein particles are retained. The total plaque burden is therefore influenced by the concentration of circulating LDL-C and other ApoB-containing lipoproteins, and by the total duration of exposure to these lipoproteins. Therefore, a person’s total atherosclerotic plaque burden is likely to be proportional to the cumulative exposure to these lipoproteins^64^. Eventually, the plaque burden and compositional changes may reach a critical point, leading to plaque disruption and formation of an overlying thrombus, which can obstruct blood flow resulting in unstable angina, myocardial infarction, or death. As a prospective study on the ApoB and coronary plaques detected by IVUS showed that multivariable analysis revealed that independently associated risk factors of progression in patients with LDL-C ≤ 70 mg/dL included baseline IVUS-derived PAV (*p* = 0.001), presence of diabetes mellitus (*p* = 0.02), increase in systolic blood pressure (*p* = 0.001), less increase in HDL-C (*p* = 0.01), and a smaller decrease in ApoB levels (*p* = 0.001)^65^. In addition, some studies have revealed that patients with high ApoB levels had longer lesion length, greater plaque volume, and a greater proportion of necrotic core as well as a lower proportion of calcified plaques in the culprit lesions using a virtual-histology IVUS compared with patients with low ApoB levels^66,67^. The association between ApoB and atheroma progression may highlight the potential importance of LDL particle concentration in patients with optimal LDL-C control. In addition, all the cholesterol within atheroma originates from ApoB particles, which enter and are trapped within the arterial wall, and ApoB particle is the basic unit of injury to the arterial wall. Thus, incorporating ApoB and Lp(a) into routine clinical care will improve assessment of cardiovascular risk due to the ApoB lipoproteins, and therapeutic intervention targeting ApoB may be able to achieve stronger lipid control.

Additionally, ApoB/ApoA-I ratio was a more sensitive predictor of the change in coronary plaque volume, than ApoB levels under strict lipid control using statins^68^. Also, an elevated ApoB/ApoA-I ratio was associated with OCT-derived presence of vulnerable plaques and thin-cap fibroatheroma^69,70^, and degree of coronary stenosis and increased non-calcified plaques detected by multidetector computed tomography^71^. ApoB/ApoA-I ratio reflects the balance between the “bad cholesterol particles and the good cholesterol particles”. Actually, the AMORIS study showed that ApoB, ApoB/ApoA-I ratio, and ApoA-I should be regarded as highly predictive in evaluation of fatal myocardial infarction risk^72^. Thus, the relationship between ApoB and ApoA-I could be of greatest value in diagnosis and treatment, and simultaneous quantitative correction of ApoB and qualitative improvement of ApoA-I through therapeutic intervention might lead to further advancement in ASCVD event avoidance.

ApoC-III, either alone or with VLDL, promotes monocytes activation and adhesion to endothelial cells, playing a causal role in the development of atherosclerotic lesions^73,74^. ApoC-III levels are positively correlated with inflammatory cytokines, including tumor necrosis factor-α and interleukin-1β^75,76^. These cytokines induce the expression of adhesion molecules such as vascular cell adhesion molecule-1, contributing to atherosclerosis and promoting calcification progression^77,78^. Also, ApoC-III increases vascular cell adhesion molecule-1 expression in human coronary artery endothelial cells; whereas, statins attenuate ApoC-III-induced monocyte adhesion^79^. As a randomized controlled trial on the ApoC-III and coronary plaques detected by NIRS-IVUS showed that both ApoC-III levels and NIRS-IVUS images at baseline and week 48 were analyzed in type 2 diabetic patients. Serial changes in IVUS-derived atheroma volume were similar between two groups (−0.7 ± 2.2 vs. −2.4 ± 1.6 mm^3^, *p* = 0.51); however, greater progression in NIRS-derived maxLCBI_4mm_ was observed in those with any increase in ApoC-III levels (91.2 ± 24.8 vs. −44.2 ± 23.5, *p* < 0.001)^80^. In addition, some studies have investigated the relationship between ApoC-III and coronary artery calcification (CAC). These studies have found that elevated ApoC-III levels are associated with increased CAC detected by CCTA^81^, and elevated ApoC-III levels are associated with severe CAC and progression to calcified nodules^82^. Considering these reports, the main cause of atherosclerotic coronary plaque formation is LDL-C, and ApoC-III may synergistically promote plaque calcification and destabilization. Thus, it should be suggested ApoC-III as a residual risk that requires therapeutic intervention added to strict LDL-C lowering therapy.

### Therapeutic advances

The current guidelines recommend that patients with ASCVD should receive LDL-C lowering therapy to target LDL-C levels <55 mg/dL, or at least <70 mg/dL in Japan^17,83,84^. LDL-C levels are mostly and directly involved in the development of atherosclerotic coronary plaques, and LDL-C lowering therapies, such as statins, ezetimibe, and proprotein convertase subtilisin/kexin 9 (PCSK9) inhibitors have contributed to coronary plaque regression^85–87^. We summarize the current clinical status of therapeutic agents targeting these apolipoproteins, as shown in Table 3.

**Table 3:** Therapeutic agents targeting each apolipoprotein

Target apolipoprotein	Therapeutic agent	Mode of action	Effect
apolipoprotein(a)	Pelacarsen	Antisense oligonucleotide inhibiting hepatic synthesis of Apo(a)	Pelacarsen therapy had significantly and substantially reduced the Lp(a) concentration in a dose-dependent manner, with mean percent changes of −80% with the 20 mg dose administered every week, −58% with the 20 mg dose administered every 2 weeks, −35% with the 20 mg dose, −56% with the 40 mg dose, −72% with the 60 mg dose administered every 4 weeks, as compared with 6% with placebo^95^.
	Olpasiran	siRNA inhibiting hepatic synthesis of Apo(a)	Olpasiran therapy had significantly and substantially reduced the Lp(a) concentration in a dose-dependent manner, resulting in placebo-adjusted mean percent changes of −70.5% with the 10 mg dose, −97.4% with the 75 mg dose, −101.1% with the 225 mg dose administered every 12 weeks, and −100.5% with the 225 mg dose administered every 24 weeks^96^.
	Zerlaciran	siRNA inhibiting hepatic synthesis of Apo(a)	Zerlaciran therapy had significantly and substantially reduced the Lp(a) concentration in a dose-dependent manner, resulting in placebo-adjusted mean percent changes of −82.8% with the 300 mg dose administered every 16 weeks, and −81.3% with the 300 mg dose and −85.6% with the 450 mg dose administered every 24 weeks^97^.
	Lepodisiran	siRNA inhibiting hepatic synthesis of Apo(a)	Lepodisiran therapy had significantly and substantially reduced the Lp(a) concentration in a dose-dependent manner, resulting in placebo-adjusted mean percent changes of −40.8% with the 16 mg dose, −75.2% with the 96 mg dose, and −93.9% with the 400 mg dose administered at baseline^98^.
	Muvalaplin (oral small molecule inhibitor)	Inhibition of Lp(a) formation by blockage of the Apo(a)–ApoB-100 interaction	Muvalaplin therapy had significantly and substantially reduced the Lp(a) concentration in a dose-dependent manner, resulting in placebo-adjusted mean percent changes of −47.6% with the 10 mg/day, −81.7% with the 60 mg/day, and −85.8% with the 240 mg/day dosage for 12 weeks, using an intact Lp(a) assay^99^.
Apolipoprotein A-I	ETC-216	Recombinant form of ApoA-I _Milano_	It has been reported that the regression of coronary atherosclerotic plaque in patients with acute coronary syndrome; the absolute reduction in atheroma volume in the combined treatment groups was −14.1 mm^3^ or a 4.2% decrease from baseline, compared with placebo group (*p* < 0.001)^104^.
	MDCO-216	Recombinant form of ApoA-I_Milano_	No significant regression of coronary atherosclerotic plaque^105^.
	CSL112	Plasma-derived ApoA-I reconstituted to form HDL-like particles	The 6 g dose of CSL112 induced a > 2-fold increase in CEC in patients after myocardial infarction. However, The AEGIS-II trial has demonstrated that four weekly infusions of CSL112 did not result in a lower risk of ASCVD than a placebo after 90 days^106,107^.
Apolipoprotein B-100	Mipomersen	Antisense oligonucleotide targeting a 20-bp segment of APOB mRNA	Lowers plasma LDL-C levels, but not available for clinical use owing to hepatotoxicity^112,115^.
Apolipoprotein C-III	Volanesorsen	Antisense oligonucleotide targeting APOC3 mRNA	Volanesorsen reduces ApoC-III levels by up to 70% in healthy individuals and 80% in patients with hyperglycemia. This is approved by the EMA for use in patients with familial chylomicronemia syndrome, but is not approved by the FDA because of the risk of thrombocytopenia^119,120^.
	Olezarsen	Antisense oligonucleotide targeting APOC3 mRNA	Olezarsen results in significantly greater reduction in triglyceride levels at 6 months than placebo among patients with moderate hypertriglyceridemia and elevated ASCVD risk. This is approved by both EMA and FDA^121,122^.
	Plozasiran	siRNA inhibiting APOC3 mRNA	Plozasiran group significantly decreased the median change from baseline in the fasting triglyceride level (−80% in the 25 mg dose, −78% in the 50 mg dose administrated every 3 months), compared with the placebo group (−17%)^123^.
	Zodasiran	siRNA inhibiting ANGPTL3 mRNA	Zodasiran has not yet received the EMA and FDA approval.
	Evinacumab	Inhibition of ANGPTL3	Evinacumab was approved in 2021 by both the EMA and FDA for the treatment of homozygous familial hypercholesterolemia^124^.

*ANGPTL3* angioproietin-related protein 3, *ApoA-I* apolipoprotein A-I, *Apo(a)* apolipoprotein(a), *ApoB-100* apolipoprotein B-100, *ApoC-II* apolipoprotein C-II, *ApoC-III* apolipoprotein C-III, *ASCVD* atherosclerotic cardiovascular disease, *CEC* cholesterol efflux capacity, *EMA* European Medicines Agency, *FDA* Federal Drug Administration, *LDL-C* low-density lipoprotein cholesterol, *Lp(a)* lipoprotein (a), *mRNA* messenger ribonucleic acid, *siRNA* small interfering ribonucleic acid.

#### Therapeutic agents targeting lipoprotein(a)

The treatments targeting Lp(a) have been gaining attention in recent years. The first Lp(a) studies in this field reported that lovastatin dose dependently increased Lp(a) levels by 33% or more^88^. The findings provided an initial indication that Lp(a), regardless of its structural similarity to LDL, may be cleared from plasma via a different pathway, and that statins may impact Lp(a) metabolism independent of its effect on LDLR. However, subsequent studies produced mixed results ranging from ineffectiveness to significant increases in Lp(a) levels with statins, raising questions regarding the consistency of findings^89,90^. In contrast, treatment with PCSK9 inhibitors reduces Lp(a)-associated Apo(a) production^91^, and contributes to reduction in NIRS-derived lipid rich plaques and CCTA-derived non-calcified necrotic plaques and pericoronary adipose tissue density^92,93^. The FOURIER trial revealed that PCSK9 inhibitors significantly reduced Lp(a) levels (median reduction 27%), and patients with higher baseline Lp(a) levels experienced greater absolute reductions and appeared to derive more pronounced coronary benefits^94^. In addition, several novel therapies, including oligonucleotide-based agents, are also in development, such as pelacarsen, olpasiran, zerlaciran, lepodisiran, and muvalaplin^95–99^. A phase II trial has shown that these agents can reduce plasma Lp(a) levels by >80–95% or more. These investigations are expected to reduce ASCVD risk and promote coronary plaque regression by targeting elevated Lp(a) levels.

#### Therapeutic agents targeting apolipoprotein A-I

Many large-scale clinical trials regarding drugs against ApoA-I have been conducted; however, there are many difficulties in applying them to clinical practice. The large-scale randomized clinical trials targeting pharmacological agents such as niacin and cholesteryl ester transfer protein inhibitors have shown that rising endogenous HDL-C does not improve cardiovascular outcomes or all-cause mortality^100–102^. These findings shifted the focus of research on HDL from quantity to quality, emphasizing the function of HDL particles rather than simply their cholesterol content. Against this background, ApoA-I, with its anti-arteriosclerotic effects, has been investigated as a therapeutic agent. One such example of this is recombinant ApoA-I Milano^103^, and ETC-216 contributed to the regression of coronary atherosclerotic plaque in patients with acute coronary syndrome^104^; whereas, MDCO-216 had a no significant regression of coronary atherosclerotic plaque^105^. Recently, CSL112, a purified wild type ApoA-I preparation from human plasma, was developed. Infusion of CSL112 enhanced LCAT activity, increased plasma HDL-C levels, accelerated cholesterol esterification, and improved CEC in patients with acute myocardial infarction^106^. The AEGIS-II trial has demonstrated that four weekly infusions of CSL112 did not result in a lower risk of myocardial infarction, stroke, or death from cardiovascular causes than a placebo after 90 days^107^. Although this trial did not meet its primary endpoint, exploratory analysis suggests that CSL112 may reduce cardiovascular death and myocardial, particularly in high-risk subgroups^108^. These findings support a potential clinical role for enhancing CEC in the management of coronary plaques.

#### Therapeutic agents targeting apolipoprotein B

The decreasing ApoB levels by statin therapy were associated with NIRS-derived lipid-rich plaques regression and IVUS-derived plaque burden regression^109,110^. Recently, the subanalysis of the HUYGENS has demonstrated that lower achieved ApoB levels (<65 mg/dL) associated with evidence of greater plaque stabilization even after controlling for LDL-C levels using PCSK9 inhibitors^111^. Although LDL-C and ApoB levels are usually closely correlated, LDL-C can underestimate ApoB in some cases, leaving residual cardiovascular risk. Therefore, when LDL-C and ApoB levels are discordant despite optimal lipid-lowering therapy, ApoB-targeted treatment targeting may be warranted. Mipomersen is a synthetic, single-stranded antisense oligonucleotide that binds the mRNA encoding ApoB-100, inhibiting its translation. Binding of mipomersen to ApoB-100 mRNA activates RNase H, which cleaves the RNA and prevents ApoB-100 protein synthesis, thereby lowering circulating ApoB levels^112^. The efficacy and safety of mipomersen have been studied in randomized clinical trials evaluating patients with homozygous familial hypercholesterolemia, heterozygous familial hypercholesterolemia, or severe hypercholesterolemia^113–115^. However, due to its mechanism of blocking VLDL lipidation and secretion, which can cause hepatic TG accumulation, the Federal Drug Administration (FDA) issued a black box warning for hepatotoxicity, and the European Medicines Agency (EMA) did not approve use of mipomersen. Thus, mipomersen is no longer available for clinical use.

#### Therapeutic agents targeting apolipoprotein C-III

While statins effectively prevent atherosclerosis through LDL-C lowering, agents targeting ApoC-III may further suppress the residual lipid-rich plaque as well as development and progression of CAC, including severe calcification and calcified nodules. In terms of pharmacological interventions for reducing ApoC-III levels, there have been reports on therapeutic agents that target TRL, such as fibrates, niacin, and omega-3 carboxylic acids. These agents have shown to reduce ApoC-III gene expression and circulating levels by between 10 and 40%^116–118^. Additionally, some studies have shown that volanesorsen, an antisense oligonucleotide that inhibits ApoC-III mRNA translation, can reduce ApoC-III levels by up to 70% in healthy individuals and 80% in patients with hyperglycemia^119,120^. Volanesorsen was approved by the EMA for use in patients with familial chylomicronemia syndrome, but was not approved by the FDA because of the risk of thrombocytopenia. While, olezarsen which is an *N*-acetylgalactosamine–conjugated antisense oligonucleotide that targets *APOC3* messenger RNA, is approved by both EMA and FDA, and it has reported that treatment with olezarsen resulted in significantly greater reduction in TG levels at 6 months than placebo among patients with moderate hypertriglyceridemia and elevated ASCVD risk^121^. In addition, it has recently reported that both olezarsen for patients with severe hypertriglyceridemia and plozasiran for patients with persistent chylomicronemia have a significantly greater reduction in the TG level and in the incidence of acute pancreatitis than placebo^122,123^. Furthermore, the therapeutic agents targeting ANGPTL3, which has emerged as novel regulators of TG and LDL-C levels in a similar way to ApoC-III, are also being developed. Evinacumab, which has inhibited ANGPTL3, was approved in 2021 by both the EMA and FDA for the treatment of homozygous familial hypercholesterolemia^124^. Zodasiran, a siRNA inhibiting ANGPTL3 mRNA, has not yet received the EMA and FDA approval (phase 3 randomized controlled trial ongoing). Previous studies have shown that lipid metabolism related to ApoC-III has a strong effect on CAC and lipid-rich plaque, so it is expected that these therapeutic agents will contribute to the correction of lesion complexity and plaque instability.

## Conclusions

We have reviewed the pathophysiological roles and genetic insights, imaging correlates, and therapeutic advance of Apo(a), ApoA-I, ApoB, and ApoC-III. The atherogenicity of Lp(a) is substantially greater than that of LDL on a per-particle basis, and elevated Lp(a) has been established as an independent risk factor for ASCVD, particularly in individuals with high LDL-C. Several novel therapies, including oligonucleotide-based agents, are in development to target elevated Lp(a), and are anticipated to provide significant clinical benefits. Although ApoA-I plays a central role in reverse cholesterol transport, and contributes to anti-atherosclerotic activity through CEC, therapeutic drugs targeting ApoA-I have not yet to be applied clinically, so we look forward to future developments. While, ApoB serves as a stronger indicator of necrotic core size and a potential biomarker for unstable plaque, compared to LDL-C. Thus, the European Society of Cardiology recommended ApoB measurement for risk assessment, particularly in patients with hypertriglyceridemia, diabetes mellitus, obesity, metabolic syndromes, or very low LDL-C. Therefore, the establishment of a treatment targeting ApoB is thought to be of great clinical significance; however, mipomersen is unfortunately no longer available for clinical use, due to critical adverse effect of hepatotoxicity. In addition, ApoC-III acts as key determinants of intravascular lipolysis and the clearance of TG-rich chylomicron and VLDL from plasma, thereby influencing atherosclerotic function as CAC and lipid core progression under strict lipid control; thus, further research into the effects of therapeutic intervention targeting ApoC-III on atherosclerotic coronary plaques and ASCVD events is anticipated; for instance, randomized trials on the effects of therapeutic agents targeting apolipoprotein C-III on coronary plaque regression and stabilization. Taken together, the remarkable progress in understanding apolipoproteins offers the potential for more comprehensive and precise strategies to prevent plaque progression and coexisting ASCVD onset.

## Data Availability

The datasets during and/or analyzed during the current study are available from the corresponding author with reasonable request.
